# Small airway function in Finnish COVID-19 survivors

**DOI:** 10.1186/s12931-021-01830-9

**Published:** 2021-08-26

**Authors:** Anna Lindahl, Jere Reijula, Leo Pekka Malmberg, Miia Aro, Tuula Vasankari, Mika Juhani Mäkelä

**Affiliations:** 1 Faculty of Medicine, University of Helsinki, Helsinki, Finland; 2 Finnish Lung Health Association, Sibeliuksenkatu 11 A 1, 00250 Helsinki, Finland; 3 Department of Pulmonology, Helsinki University Hospital, Helsinki, Finland; 4 Skin and Allergy Hospital, University of Helsinki and Helsinki University Hospital, Helsinki, Finland; 5 Faculty of Medicine, Department of Pulmonary Diseases and Clinical Allergology, University of Turku, Turku, Finland

**Keywords:** COVID-19, Small airways, Airway inflammation, Viral infection, Nitric oxide, Impulse oscillometry

## Abstract

Follow-up studies of COVID-19 patients have found lung function impairment up to six months after initial infection, but small airway function has not previously been studied. Patients (n = 20) hospitalised for a severe SARS-CoV-2 infection underwent spirometry, impulse oscillometry, and multiple measurements of alveolar nitric oxide three to six months after acute infection. None of the patients had small airway obstruction, nor increased nitric oxide concentration in the alveolar level. None of the patients had a reduced FEV_1_/FVC or significant bronchodilator responses in IOS or spirometry. In conclusion, we found no evidence of inflammation or dysfunction in the small airways.

## Introduction

There is some knowledge already emerging on the effects of Coronavirus Disease 2019 (COVID-19) on lung function. Lung function impairment has been described up to six months after acute infection [1, 2]. To date, these studies have indicated that COVID-19 infection affects mostly lung volume and diffusing capacity. In addition, follow-up studies of the previous coronavirus diseases Severe Acute Respiratory Syndrome (SARS) and Middle East Respiratory Syndrome (MERS) have shown persisting pulmonary impairment, including ventilation restriction and impaired diffusing capacity, as well as lung fibrosis and other radiological abnormalities, persisting in up to 15 years follow-up [3, 4]. To our knowledge, small airway function measurements, such as impulse oscillometry (IOS) or extended exhaled nitric oxide (NO) measurements, have not previously been studied in SARS, MERS, or COVID-19 patients.

## Methods

Patients hospitalised due to a documented COVID-19 in the Helsinki metropolitan area, covered by Helsinki University Hospital in Finland, underwent lung function tests three to six months after hospital discharge. The inclusion criteria were (a) the patient had been admitted to the intensive care unit (ICU) or (b) the cohort ward clinician had classified the patient’s disease as “severe”. Twenty-seven patients were randomly selected, according to the capacity of the routine clinical physiology lab’s resources, to undergo IOS and multiple flow measurements of exhaled NO in addition to spirometry and diffusing capacity test. Five patients later declined to participate or did not give written consent, and two patients were later excluded for use of inhaled long-acting beta agonist therapy. The study was approved by the Research Ethics Committee of Helsinki University Hospital (§148 HUS/1922/2020).

IOS and spirometry manoeuvres were measured in this particular order, and triplicate measurements were recorded in line with ATS/ERS guidelines using Vyntus Pneumo/IOS (Vyaire Medical, Hoechberg, Germany). Participants inhaled 400 μg salbutamol via spacer (Volumatic) immediately after the baseline measurements and performed postbronchodilator measurements 15 min later. Spirometry variables evaluated were forced vital capacity (FVC), forced expiratory volume in one second (FEV_1_), forced expiratory ratio (FEV_1_/FVC), maximal mid-expiratory flow (MMEF), and maximal flow at 50% of FVC (MEF_50_). IOS variables evaluated were resistance at 5 Hz (R5), the frequency dependence of resistance in terms of the difference between R5 and resistance at 20 Hz (R5-20), reactance at 5 Hz (X5), and area of reactance (AX).

Multiple flow measurements of exhaled NO (30, 50, 100 and 200 ml/s) were evaluated with a chemiluminescence analyser (CLD 77, EcoPhysics, Duernten, Switzerland) to obtain the fractional concentration of NO with flow 50 ml/s (FE_NO_), and the alveolar NO concentration (NO_alv_) by using the extended analysis [5].

The lung function results were compared with the healthy reference values of the same gender, age and/or height and expressed as Z-scores [6–9].

## Results

The mean age of participants (14/20 male) was 56 (range 34–72), and the mean BMI was 29.6. Ten patients (50%) had one or more comorbidities, the two most common being hypertension (30%) and type 2 diabetes (25%). One patient had asthma but did not use any inhaled asthma medications. Other pulmonary diseases were not present in the study population. There were no current smokers in the study population, while six participants (30%) reported former smoking, 13 (70%) were never-smokers, and one gave no answer.

The mean duration of hospital treatment was 19 days. Ten (50%) were admitted to ICU and eight (40%) needed mechanical ventilation. The mean duration of intensive care was 17 days and mechanical ventilation 16 days, respectively. Of the 12 participants who were not intubated, 10 (91%) needed supplementary oxygen therapy, with the mean peak flow rate of 10 l/min. Three (15%) had a pulmonary embolism during initial hospitalisation or during the recovery.

**Fig. 1:**
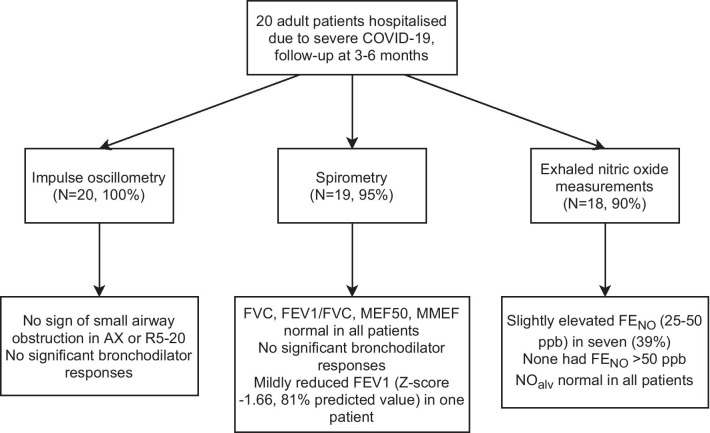
Flowchart of small airway function at 3–6 months follow-up in 20 adult patients hospitalised due to COVID-19. *AX* area of reactance, *R5-20* differential change in airway resistance between 5 and 20 Hz, *FVC* forced vital capacity, *FEV1/FVC* forced expiratory ratio, *MEF50* maximal flow at 50% of FVC, *MMEF* maximal mid-expiratory flow, *FEV1* forced expiratory volume in 1 s, *FE*_*NO*_ fractional concentration of nitric oxide with flow 50 ml/s, *NO*_*alv*_ alveolar nitric oxide concentration

IOS, spirometry, and exhaled NO measurements were performed by 20 (100%), 19 (95%), and 18 (90%) participants, respectively. On average, the lung function tests were performed 154 days (5.1 months) after symptom onset. The results are presented in Table 1 and summarised in Fig. 1.

**Table 1:** Spirometry, impulse oscillometry and exhaled nitric oxide results

	Pre-bronchodilator		Post-bronchodilator		Change
	Abs	Z	Abs	Z	%
Spirometry					
FVC, *l*					
Mean ± SD	4.53 ± 0.96	− 0.18 ± 0.88	4.45 ± 1.01	− 0.30 ± 0.96	− 1.8 ± 2.3
Median (IQR)	4.47 (3.99–4.88)	− 0.10 (− 0.87–0.24)	4.28 (3.87–5.02)	− 0.13 (− 1.09–0.28)	− 2.4 (− 3.5–0.0)
Range	2.45–6.46	− 1.28–1.65	2.39–6.54	− 1.67–1.78	− 5.3–2.9
FEV_1_, *l*					
Mean ± SD	3.59 ± 0.71	0.09 ± 0.91	3.65 ± 0.74	0.21 ± 0.94	1.7 ± 1.9
Median (IQR)	3.55 (3.23–3.99)	0.23 (− 0.59–0.73)	3.63 (3.21–4.15)	0.31 (− 0.44–0.92)	2.2 (− 0.3–3.3)
Range	1.96–5.28	− 1.66–2.15	1.97–5.37	− 1.62–2.31	− 2.5–4.7
FEV_1_/FVC, %					
Mean ± SD	79.57 ± 4.24	0.57 ± 0.99	82.45 ± 4.89	1.11 ± 1.06	3.6 ± 2.1
Median (IQR)	81.55 (75.78–82.72)	0.89 (− 0.43–1.39)	82.34 (79.25–85.97)	0.91 (− 0.07–2.15)	3.2 (2.1–5.8)
Range	68.88–84.62	− 0.93–1.75	71.10–90.53	− 0.52–2.62	− 0.1–7.0
MMEF, *l*·s^−1^					
Mean ± SD	3.49 ± 0.95	0.49 ± 1.13	3.95 ± 1.03	1.02 ± 1.23	13.6 ± 6.6
Median (IQR)	3.49 (2.68–4.09)	0.59 (− 0.41–1.06)	3.99 (3.04–4.77)	0.94 (− 0.07–1.79)	13.4 (9.3–16.0)
Range	1.87–5.57	− 1.48–2.81	2.07–6.06	− 0.95–3.32	− 0.9–26.7
MEF_50_, *l*·s^−1^					
Mean ± SD	4.23 ± 1.10	0.44 ± 1.04	4.83 ± 1.07	0.99 ± 0.97	15.9 ± 14.3
Median (IQR)	4.02 (3.46–4.91)	0.61 (− 0.21–1.00)	4.78 (3.98–5.63)	1.25 (0.37–1.42)	13.2 (7.5–19.0)
Range	2.21–6.54	− 1.28–2.45	2.78–7.03	− 0.76–2.86	− 10.4–49.9
IOS					
R5, kPa·*l*^−1^·s					
Mean ± SD	0.30 ± 0.09	− 0.06 ± 1.00	0.27 ± 0.07	− 0.60 ± 1.58	− 7 ± 12
Median (IQR)	0.28 (0.24–0.39)	− 0.01 (− 0.51–0.38)	0.27 (0.23–0.32)	− 0.29 (− 0.95–0.04)	− 8 (− 15–3)
Range	0.13–0.45	− 1.89–2.49	0.12–0.39	− 5.78–1.98	− 23–18
R5-20, kPa·*l*^−1^·s					
Mean ± SD	0.03 ± 0.03	− 0.98 ± 0.98	0.03 ± 0.02	− 1.04 ± 1.55	13 ± 73
Median (IQR)	0.04 (0.01–0.05)	− 0.79 (− 1.55 to − 0.45)	0.02 (0.01–0.06)	− 0.86 (− 1.73 to − 0.42)	− 10 (− 42–60)
Range	− 0.02–0.09	− 2.94–1.11	− 0.02–0.06	− 4.41–3.58	− 95–179
X5, kPa·*l*^−1^·s					
Mean ± SD	− 0.08 ± 0.03	1.07 ± 1.39	− 0.07 ± 0.03	1.69 ± 2.00	− 16 ± 21
Median (IQR)	− 0.08 (− 0.09 to − 0.06)	0.76 (0.05–1.81)	− 0.07 (− 0.08 to − 0.05)	1.08 (0.54–2.19)	− 16 (− 31–1)
Range	− 0.14 to − 0.03	− 0.74–5.05	− 0.13 to − 0.01	− 0.99–7.10	− 59–21
AX, kPa·L^−1^					
Mean ± SD	0.27 ± 0.19	− 0.32 ± 0.66	0.19 ± 0.13	− 0.10 ± 1.52	− 22 ± 30
Median (IQR)	0.29 (0.10–0.41)	− 0.18 (− 0.60–0.11)	0.18 (0.08–0.29)	− 0.37 (− 0.63 to − 0.19)	− 26 (− 46 to − 6)
Range	0.04–0.72	− 2.46–0.67	0.01–0.46	− 1.22–6.13	− 75–53
Exhaled NO					
FE_NO_, ppb					
Mean ± SD	21.7 ± 6.9	N/A	N/A	N/A	N/A
Median (IQR)	21.8 (17.0–28.2)	N/A	N/A	N/A	N/A
Range	9.1–31.2	N/A	N/A	N/A	N/A
NO_alv_, ppb					
Mean ± SD	1.4 ± 1.0	− 1.19 ± 0.58	N/A	N/A	N/A
Median (IQR)	1.4 (0.6–1.7)	− 1.20 (− 1.69 to − 1.02)	N/A	N/A	N/A
Range	0.2–3.9	− 1.93–0.30	N/A	N/A	N/A

*FVC* forced vital capacity, *FEV*_*1*_ forced expiratory volume in 1 s, *FEV*_*1*_*/FVC* forced expiratory ratio, *MMEF* maximal mid-expiratory flow, *MEF*_*50*_ maximal flow at 50% of FVC, *IOS* impulse oscillometry, *R5* resistance at 5 Hz, *R5-20* differential change in airway resistance between 5 and 20 Hz, *X5* reactance at 5 Hz, *AX* area of reactance, *NO* nitric oxide, *FE*_*NO*_ fractional concentration of NO with flow 50 ml/s, *NO*_*alv*_ alveolar NO concentration

There was no evidence of small airway obstruction in IOS in any of the 22 participants, as expressed by the frequency dependence of resistance (R5-20) or the reactance area (AX), which are putative markers of small airway function [10]. One patient had a mildly reduced FEV_1_ (z = − 1.66, 81% of predicted). The patients had neither a lowered FEV_1_/FVC, MMEF, MEF_50_, nor a significant bronchodilator response in spirometry or in IOS (R5 reduction > 40%).

Seven patients (39%) had slightly elevated FE_NO_ of 25 to 50 ppb, indicating possible inflammation*.* None of the participants had an abnormal FE_NO_ of more than 50 ppb. In addition, NO_alv_, indicating the NO production in the small airways, was low or in the normal range in all participants (Table 1).

## Discussion

Our study indicates that even among patients with very severe COVID-19 infection, the bronchial inflammation distally in the small airways is not present three to six months after original infection, and there were neither long-term impairments in small airway function, nor new asthma cases. This could imply that COVID-19 does not induce chronic bronchial inflammation or predispose to chronic obstructive diseases.

One should note that our sample size was small due to the clinical physiology lab’s limited resources amidst the pandemic, and our study lacked a control group. However, the assessment of small airway indices was based on previously published reference values matched with the present study sample. Moreover, the participants in our study had recovered from a considerably severe COVID-19, with half of the patients (10/20) admitted to ICU and almost half (8/20) needing mechanical ventilation due to COVID-19. Thus, our results cannot be directly generalised to patients with a milder disease, although the severity of lung function impairment has previously been associated with the severity of the initial infection [1], suggesting that patients with a less severe COVID-19 might have even fewer abnormal findings in small airway function.

In conclusion, small airways do not seem to be affected in COVID-19 survivors at three to six months after the initial infection. Therefore, future follow-up studies should focus on the effects of COVID-19 on diffusing capacity and lung volumes, as evidence suggests that COVID-19 causes damage rather to the lung parenchyma and microcirculation than on the bronchial level.

## Data Availability

Data are available upon reasonable request.

## Abbreviations

AX: Area of reactance
FEV_1_: Forced expiratory volume in 1 s
FE_NO_: Fractional concentration of nitric oxide with flow 50 ml/s
FEV_1_/FVC: Forced expiratory ratio
FVC: Forced vital capacity
ICU: Intensive care unit
IOS: Impulse oscillometry
MEF_50_: Maximal flow at 50% of forced vital capacity
MERS: Middle East respiratory syndrome
MMEF: Maximal mid-expiratory flow
NO: Nitric oxide
NO_alv_: Alveolar nitric oxide concentration
R5: Resistance at 5 Hz
R5-20: Differential change in airway resistance between 5 and 20 Hz
SARS: Severe acute respiratory syndrome
X5: Reactance at 5 Hz
